# Development and internal validation of a clinical prediction model for septic shock in pediatric respiratory syncytial virus bronchiolitis based on routine blood biomarkers and concomitant fungal infection

**DOI:** 10.3389/fcimb.2026.1743976

**Published:** 2026-05-15

**Authors:** Junyu Dong, Yikang Ouyang, Jingwen Ni, Mengxin Zhao, Zhihui Du, Kenan Fang

**Affiliations:** 1 Pediatric Intensive Care Unit, Luoyang Maternal and Child Health Hospital, Luoyang, Henan, China; 2 Intensive Care Unit, Clinical Medical College & Affiliated Hospital of Chengdu University, Chengdu, Sichuan, China

**Keywords:** bronchiolitis, pediatric intensive care unit, prediction model, respiratory syncytial virus, septic shock

## Abstract

**Background:**

Respiratory syncytial virus (RSV) bronchiolitis is a leading cause of pediatric intensive care unit (PICU) admissions worldwide, with septic shock representing a severe and potentially fatal complication. Early identification of high-risk patients remains challenging in clinical practice. This study aimed to develop and validate a reliable prediction model for septic shock in children admitted to PICU with RSV bronchiolitis using readily available clinical and laboratory parameters.

**Methods:**

A retrospective cohort study was conducted on 224 pediatric patients with RSV bronchiolitis admitted to PICU. Demographic characteristics, clinical features, and laboratory findings upon admission were systematically collected. Given the low event rate, Firth logistic regression was employed for univariate and multivariate analyses to mitigate small-sample bias. Model performance was rigorously evaluated using 5-fold cross-validation, bootstrap resampling, calibration metrics (Hosmer-Lemeshow test, Brier score, calibration intercept and slope), and discrimination measures (area under the receiver operating characteristic curve). Development of web calculator based on Shiny app to facilitate clinical implementation.

**Results:**

Four independent predictors of septic shock were identified through multivariate Firth logistic regression: fungal co-infection (adjusted odds ratio [aOR]: 9.01, 95% confidence interval [CI]: 2.26-36.49, *P* = .003), elevated admission glucose (aOR: 1.23, 95% CI: 1.04-1.48, *P* = .02), decreased antithrombin III (aOR: 0.96, 95% CI: 0.94-0.99, *P* = .004), and elevated interleukin-6 (aOR: 1.00, 95% CI: 1.00-1.01, *P* = .008). The final model demonstrated excellent discrimination with an area under the curve (AUC) of 0.892 (95% CI: 0.870-0.914) in 5-fold cross-validation and 0.906 (95% CI: 0.838-0.949) in bootstrap validation. Calibration was optimal (Hosmer-Lemeshow χ²=5.991, *P* = .648; Brier score=0.065; calibration slope=0.957; calibration intercept=0.002). At the optimal threshold of 0.154, the model achieved sensitivity of 75.0%, specificity of 90.5%, and overall accuracy of 88.8%.

**Conclusions:**

We developed and internally validated a prediction model incorporating fungal co-infection, admission glucose, antithrombin III, and interleukin-6 for early identification of septic shock risk in pediatric RSV bronchiolitis patients. The model demonstrated good discrimination (AUC 0.892) in this single-center cohort. However, given the modest sample size, external validation in larger, multicenter populations is essential before clinical implementation. This tool may support early risk stratification and clinical decision-making, though its impact on patient outcomes requires further evaluation.

**Clinical trial registration:**

http://www.chictr.org.cn/index.aspx, identifier ChiCTR2200057182.

## Introduction

Respiratory syncytial virus (RSV) is a leading cause of lower respiratory tract infection requiring pediatric intensive care unit (PICU) admission (Kang et al., 2019; Halasa et al., 2023; Bender et al., 2024; Phijffer et al., 2025). While most children recover without major complications, a subset of critically ill RSV patients progress rapidly to septic shock, resulting in multiorgan dysfunction and increased mortality (Byington et al., 2015; Geoghegan et al., 2017).Despite this significant clinical impact, tools for early identification of children at highest risk of RSV-associated septic shock are currently lacking, leaving clinicians unable to proactively stratify risk at the time of ICU admission (Watson et al., 2024).

Existing pediatric septic and shock prediction tools—such as the Pediatric Sequential Organ Failure Assessment (pSOFA) and the Septic Prevalence, Outcomes, and Therapies (SPOT) score—have largely been developed from heterogeneous infectious cohorts and often rely on dynamic physiologic and laboratory data collected after PICU admission, limiting their utility for guiding immediate intervention at ICU entry (Balamuth et al., 2022; García-Mauriño and Bassat, 2024; Malik et al., 2024). In RSV patients specifically, septic shock frequently manifests as a non-classical “cold shock” phenotype, with microcirculatory impairment and early tissue hypoperfusion preceding overt hypotension, while vital signs may remain superficially stable due to strong pediatric compensatory mechanisms (Top et al., 2011; Moore et al., 2015; Kuiper et al., 2016). Routine laboratory markers, including C-reactive protein and procalcitonin, confirm infection but do not reliably predict progression to shock (Daud et al., 2024; Pérez et al., 2024). This temporal gap between underlying deterioration and clinical recognition may result in missed opportunities for timely intervention, increasing the risk of organ dysfunction and mortality.

To address this gap, we aimed to develop and validate a multivariable prediction model using only baseline clinical and laboratory parameters available at PICU admission. The goal is to provide a clinically applicable tool for early risk stratification of RSV-infected children, which may facilitate early identification of high-risk patients and support closer monitoring and informed clinical decision-making at the time of PICU admission.

## Methods

### Study design and population

This retrospective, single-center cohort study included pediatric patients admitted to the PICU with laboratory-confirmed RSV infection between January 1, 2023, and September 30, 2025, at Luoyang Maternal and Child Health Hospital (Henan, China). The study was conducted in accordance with the principles of the Declaration of Helsinki and was approved by the Medical Ethics Committee of Luoyang Maternal and Child Health Hospital (Ethical approval number: KY2022021401.0). The study protocol was registered with the Chinese Clinical Trial Registry (ChiCTR2200057182). Given the retrospective design and use of anonymized clinical data, the requirement for informed consent was waived by the ethics committee. This study was reported in accordance with the STROBE and TRIPOD statements, and all methods were performed in accordance with relevant guidelines and regulations.

### Study population

Inclusion criteria: 1) Age between 29 days and 18 years; 2) Laboratory-confirmed RSV infection via nasopharyngeal aspirate or swab using multiplex PCR or RSV-specific RT-PCR (**Supplementary Digital Content 1**); 3) Admission to the PICU for RSV-associated acute lower respiratory tract infection, including bronchiolitis or pneumonia, requiring respiratory support or intensive monitoring; 4) Complete baseline demographic, clinical, and laboratory data recorded within 24 hours of admission, with ≤10% missing values for key predictive variables; and 5) Clearly documented clinical outcomes during the PICU stay. Exclusion Criteria: 1) RSV infection occurring ≥48 hours after hospital admission; 2) Known or suspected primary or secondary immunodeficiency, including HIV infection, chemotherapy-induced immunosuppression, or prior organ or hematopoietic stem cell transplantation; and 3) Transfer from another institution with ICU care exceeding 24 hours, to ensure that baseline data accurately reflected the initial admission status.

### Outcome definition

The outcome was development of septic shock during PICU hospitalization, defined according to the Surviving Septic Campaign pediatric guidelines as suspected or proven infection accompanied by cardiovascular dysfunction requiring vasoactive-inotropic support to maintain adequate perfusion despite adequate fluid resuscitation (Weiss et al., 2020). Septic shock diagnosis was ascertained through comprehensive review of medical records, including documentation of hemodynamic instability, lactate elevation, vasoactive medication administration, and physician clinical diagnosis.

### Data collection

Comprehensive data were systematically extracted from electronic medical records by trained research personnel using standardized case report forms. Variables collected included: (1) Demographic; (2) Clinical characteristics; (3) Infection profiles; (4) Laboratory parameters upon PICU admission; and (5) Clinical course.

Fungal co-infection was defined by positive microbiological testing (culture, PCR, or antigen detection) from respiratory specimens obtained within 24 hours of PICU admission, with results available to clinicians at the time of initial assessment or within the first hospital day. In our institution, preliminary microbiological data (e.g., Gram stain, fungal antigen tests, or rapid PCR) are often available within 24 hours for high-risk patients, allowing for early clinical suspicion and initial risk stratification while awaiting definitive culture results.

### Missing data

Missing data were handled using multiple imputation by chained equations (MICE) with five imputed datasets. The first completed dataset was used for statistical analysis.

### Statistical analysis

Continuous variables were assessed for normality using the Shapiro-Wilk test. Variables with normal and non-normal distributions were reported as mean (standard deviation, SD) and median [interquartile range, IQR], respectively, and were compared between groups using the Mann-Whitney U test. Categorical variables were expressed as frequency (percentage) and compared using the chi-square or Fisher’s exact test, as appropriate. Standardized mean differences (SMD) were calculated to evaluate covariate balance, with SMD >0.1 indicating meaningful imbalance.

Given the low incidence of septic shock in pediatric RSV bronchiolitis populations, Firth logistic regression was applied for both univariate and multivariate analyses to reduce small-sample bias and enhance coefficient stability (D'Angelo and Ran, 2025). This method relaxed the conventional event-per-variable (EPV) ≥10 requirement, making it particularly appropriate for rare-event data. Univariate Firth logistic regression was first performed for all candidate predictors to identify those associated with septic shock (*P* <.05). Predictors showing significance in univariate analysis were then entered into multivariate Firth logistic regression, and factors with *P* <.05 in the multivariate model were retained as the final predictors.

We additionally performed LASSO (least absolute shrinkage and selection operator) logistic regression as a sensitivity analysis to assess the robustness of variable selection. LASSO regression with 10-fold cross-validation was employed to select the optimal penalty parameter λ, balancing model complexity and goodness of fit.

To assess the robustness of the model and address concerns regarding the timeliness of fungal co-infection as an admission predictor, we performed a sensitivity analysis by refitting the multivariate Firth logistic regression model after excluding the fungal co-infection variable. The performance of this reduced model was evaluated using the same internal validation procedures (5-fold cross-validation and bootstrap resampling) and metrics (AUC, calibration, sensitivity, specificity, positive predictive value, negative predictive value, and accuracy) as the original model.

Model discrimination was evaluated using the area under the receiver operating characteristic curve (AUC). Internal validation was conducted using two complementary strategies: 1) 5-fold cross-validation repeated five times to estimate out-of-sample performance, and 2) bootstrap resampling (1,000 iterations) with bias-corrected AUC estimates and 95% confidence intervals. Model calibration was assessed using the Hosmer-Lemeshow goodness-of-fit test (10 groups), Brier score, calibration intercept (ideal: 0), and calibration slope (ideal: 1). Calibration plots were generated to visualize agreement between predicted probabilities and observed outcomes.

The optimal probability threshold was determined using Youden’s index. At this threshold, sensitivity, specificity, positive predictive value, negative predictive value, accuracy, and F1 score were calculated. A web-based risk calculator was developed using the Shiny package in R, implementing the final multivariate model to facilitate real-time bedside risk assessment. The calculator included input fields for the final key predictors and provided instantaneous risk probabilities with clinical interpretation.

All statistical analyses were performed using R software version 4.5.1 (R Foundation for Statistical Computing, Vienna, Austria). Two-tailed *P* <.05 was considered statistically significant.

## Results

### Patient recruitment and flow

A total of 287 hospitalized pediatric patients aged between 29 days and 18 years with laboratory-confirmed RSV infection were initially assessed for eligibility. After applying the exclusion criteria, 18 patients were excluded due to nosocomial RSV infection (≥48 hours after admission), 12 patients due to known or suspected immunodeficiency (HIV, chemotherapy, or transplantation), 21 patients due to transfer from another institution with more than 24 hours of ICU care, and 12 patients due to incomplete baseline data (>10% missing values for key variables). The final analysis cohort thus consisted of 224 pediatric patients, of whom 24 (10.7%) developed septic shock during their PICU stay and 200 (89.3%) did not. The patient selection process is systematically summarized in **Supplementary Digital Content 2**.

### Missing data and baseline characteristics

The proportion of missing values for each variable and the method of filling them are presented in **Supplementary Digital Content 3**. A total of 224 pediatric patients were included. Missingness was low, mainly affecting glucose (5.36%), fungal infection (1.79%), and procalcitonin (1.34%). Little’s MCAR test (χ²=5190, df=5928; p=1.000) indicated data were missing completely at random. Missing data were handled using multiple imputation by chained equations, with models selected by variable type (predictive mean matching for continuous variables, logistic regression for binary variables, and multinomial logistic regression for categorical variables). Five imputed datasets were generated (50 iterations; seed=123), and the first completed dataset was used for analysis.

A total of 224 pediatric patients with RSV infection requiring PICU admission were included in the final analysis, of whom 24 (10.7%) developed septic shock. The median age was 6.84 months [IQR: 3.06–12.44 months], and 78 (34.8%) were female. Baseline characteristics stratified by septic shock status are presented in **Table 1**. Compared to patients without septic shock, those who developed shock had significantly higher rates of fungal co-infection (25.0% vs. 3.1%, P < 0.001) and congenital heart disease (16.7% vs. 5.5%, P = 0.102), as well as more frequent antibiotic escalation (45.5% vs. 25.3%, P = 0.133). At admission, the shock group exhibited marked metabolic and inflammatory disturbances, including elevated glucose (median 8.45 vs. 6.40 mmol/L, P < 0.001), reduced antithrombin III (median 79.80 vs. 103.65 g/L, P < 0.001), and higher levels of interleukin-6 (median 44.95 vs. 14.48 pg/mL, P = 0.012). Other inflammatory markers, including C-reactive protein and procalcitonin, were also significantly elevated in the shock group (all P ≤ 0.010). Hemoglobin levels were markedly lower in the shock group (median 92.0 vs. 109.0 g/L, P < 0.001), and coagulation abnormalities such as prolonged prothrombin time and elevated D-dimer were more pronounced (all P ≤ 0.005). Consequently, patients who developed septic shock experienced substantially longer PICU stays (median 22.5 days [IQR: 17.75–27.50] vs. 10.0 days [IQR: 8.00–13.00], P < 0.001).

**Table 1:** Baseline clinical and demographic characteristics of the study population.

Variable	No septic shock (n=200)	Septic shock (n=24)	*P* value	SMD
Demographics				
Female	68 (34.0%)	10 (41.7%)	.602	0.16
Age, months	6.92 [3.12, 12.25]	6.24 [2.36, 13.50]	.483	0.06
Allergy History	23 (11.7%)	2 (8.7%)	.944	0.10
Asthma History	39 (19.6%)	3 (12.5%)	.571	0.19
Congenital Heart Disease	11 (5.5%)	4 (16.7%)	.102	0.36
Clinical Features				
High Dependency	8 (4.0%)	0 (0.0%)	.680	0.29
Fever	118 (59.0%)	18 (75.0%)	.203	0.35
Wheezing	151 (75.5%)	16 (66.7%)	.490	0.20
Infections				
Bacterial Infection	113 (56.5%)	17 (70.8%)	.262	0.30
Viral Infection	72 (36.0%)	8 (33.3%)	.971	0.06
Fungal Infection	6 (3.1%)	6 (25.0%)	<.001	0.67
Antibiotics Use			.133	0.44
None	9 (4.5%)	1 (4.5%)		
Used	139 (70.2%)	11 (50.0%)		
Upgraded	50 (25.3%)	10 (45.5%)		
Length of Stay	10.00 [8.00, 13.00]	22.50 [17.75, 27.50]	<.001	1.75
Admission Glucose, mmol/L	6.40 [5.60, 7.30]	8.45 [7.35, 11.07]	<.001	0.79
Blood Routine				
Neutrophil Count, ×10^9^/L	4.04 [2.27, 6.29]	4.65 [2.54, 7.58]	.402	0.34
Lymphocyte Count, ×10^9^/L	3.87 [2.27, 5.77]	2.64 [1.54, 5.73]	.290	0.04
Platelet, ×10^9^/L	406.00 [314.50, 534.50]	395.50 [313.75, 464.00]	.464	0.23
White blood cell count, 10^9^/L	9.68 [7.08, 12.18]	10.11 [5.83, 18.49]	.732	0.16
Hemoglobin, g/L	109.00 [99.00, 118.25]	92.00 [84.75, 102.25]	<.001	0.92
NLR	1.19 [0.48, 2.14]	1.52 [0.75, 3.19	.212	0.29
Inflammatory Markers				
C-reactive protein, mg/L	2.16 [0.50, 9.68]	7.36 [1.88, 24.91]	.010	0.40
Procalcitonin, ng/mL	0.17 [0.11, 0.48]	0.36 [0.21, 2.08]	.002	0.51
IL-6, pg/mL	14.48 [3.45, 29.78]	44.95 [7.88, 176.78]	.012	0.47
Ferritin, μg/mL	108.46 [59.83, 190.80]	136.50 [97.71, 297.13]	.053	0.54
Organ Function				
BUN, mmol/L	2.66 [1.85, 3.73]	3.90 [2.62, 4.68]	.010	0.03
Creatinine, μmol/L	21.30 [17.38, 25.63]	20.40 [16.12, 23.85]	.482	0.05
Uric Acid, μmol/L	228.50 [183.93, 294.22]	204.95 [151.05, 357.30]	.893	0.22
AST, U/L	40.00 [33.00, 54.00]	40.00 [31.00, 60.75]	.872	0.06
ALT, U/L	24.00 [18.00, 32.00]	24.00 [14.75, 35.00]	.794	0.01
Myocardial Injury Markers				
CK, U/L	94.00 [62.75, 129.50]	71.00 [32.25, 101.68]	.122	0.39
CK-MB, U/L	25.00 [19.00, 34.00]	25.50 [12.75, 36.25]	.673	0.10
LDH, U/L	307.50 [270.50, 351.32]	358.45 [309.50, 412.50]	.005	0.62
Coagulation Function				
PT, second	11.40 [10.80, 12.60]	12.20 [11.50, 13.98]	.005	0.55
APTT, second	30.50 [26.87, 35.10]	33.65 [26.18, 40.33]	.383	0.31
D-Dimer, μg/mL	0.42 [0.26, 0.62]	0.69 [0.42, 1.48]	.002	0.45
AT3, g/L	103.65 [93.45, 110.50]	79.80 [70.40, 102.47]	<.001	0.88
Electrolytes				
Potassium, mmol/L	4.66 [4.20, 5.06]	4.36 [3.93, 4.83]	.093	0.33
Sodium, mmol/L	138.10 [136.20, 139.83]	136.45 [135.67, 138.65]	.144	0.06
Calcium, mmol/L	2.35 [2.26, 2.46]	2.29 [2.20, 2.37]	.043	0.13

IL-6, interleukin-6; LDH, lactate dehydrogenase; BUN, blood urea nitrogen; PT, prothrombin time; AT3, antithrombin III; CK, creatine kinase. P <.05 means statistically significant; SMD: Standardized Mean Difference, SMD > 0.1 means Balanced Difference; Median [IQR] was used for continuous variables, categorical variables used Frequency (percentage); Tests: chi-square test for categorical variables, non-parametric test for continuous variables.

### Univariate analysis of risk factors

Univariate Firth logistic regression identified multiple factors significantly associated with septic shock, including congenital heart disease(OR: 3.62, 95% CI: 1.01–11.25, *P* <.05), fungal co-infection (OR: 10.51, 95% CI: 3.15–35.37, *P* <.001), admission glucose (OR: 1.33, 95% CI: 1.16–1.54, *P* <.001), hemoglobin (OR: 0.94, 95% CI: 0.91–0.97, *P* <.001), procalcitonin (OR: 1.24, 95% CI: 1.07–1.58, *P* <.001), IL-6 (OR: 1.00, 95% CI: 1.00–1.01, *P* <.001), ferritin (OR: 1.00, 95% CI: 1.00–1.01, *P* = .001), lactate dehydrogenase (OR: 1.01, 95% CI: 1.00–1.01, *P* = .001), prothrombin time (OR: 1.21, 95% CI: 1.03–1.52, *P* = .021), D-dimer (OR: 1.17, 95% CI: 1.05–1.34, *P* = .005), antithrombin III (OR: 0.96, 95% CI: 0.93–0.98, *P* <.001), and creatine kinase (OR: 1.00, 95% CI: 1.00–1.00, *P* = .005) (**Table 2**).

**Table 2:** Univariate and firth-adjusted ratio analyses of risk factors for septic shock on admission to PICU.

Variable	Unadjusted OR (95% CI)	*P* value	Firth-adjusted OR (95% CI)	*P* value
Demographics				
Female	1.40 (0.59-3.25)	.453	–	–
Age, months	1.00 (0.97-1.02)	.992	–	–
Allergy History	0.84 (0.16-2.84)	.803	–	–
Asthma History	0.65 (0.17-1.88)	.450	–	–
Congenital Heart Disease	3.62 (1.01-11.25)	.052	1.82 (0.21-12.40)	.561
Clinical Features				
High Dependency	0.46 (0.004-3.91)	.563	–	–
Fever	1.98 (0.81-5.46)	.144	–	–
Wheezing	0.63 (0.27-1.61)	.323	–	–
Infections				
Bacterial Infection	1.80 (0.76-4.68)	.192	–	–
Viral Infection	0.91 (0.36-2.15)	.841	–	–
Fungal Infection	10.51 (3.15-35.37)	**<.001**	9.01 (2.26-36.49)	**.003**
Antibiotics				
Used	0.67 (0.14-6.56)	.678	–	–
Upgraded	1.46 (0.29-14.51)	.679	–	–
Admission Glucose, mmol/L	1.33 (1.16-1.54)	**<.001**	1.23 (1.04-1.48)	**.022**
Blood Routine				
Neutrophil Count, ×10^9^/L	1.08 (0.99-1.17)	.072	–	–
Lymphocyte Count, ×10^9^/L	1.01 (0.88-1.10)	.910	–	–
Platelet, ×10^9^/L	1.00 (1.00-1.00)	.891	–	–
White blood cell count, ×10^9^/L	1.02 (0.98-1.05)	.323	–	–
Hemoglobin, g/L	0.94 (0.91-0.97)	**<.001**	0.97 (0.93-1.00)	.082
NLR	1.14 (0.96-1.33)	.120	–	–
Inflammatory Markers				
C-reactive protein	1.01 (1.00-1.02)	.061	–	–
Procalcitonin, ng/mL	1.24 (1.07-1.58)	**<.001**	0.98 (0.88-1.33)	.721
IL-6, pg/mL	1.00 (1.00-1.01)	**<.001**	1.00 (1.00-1.01)	**.008**
Ferritin, μg/mL	1.00 (1.00-1.01)	**<.001**	1.00 (1.00-1.00)	.830
Organ Function				
BUN, mmol/L	1.01 (0.99-1.02)	.429	–	–
Creatinine, μmol/L	1.00 (0.99-1.01)	.422	–	–
Uric Acid, μmol/L	1.00 (1.00-1.01)	.161	–	–
AST, U/L	1.00 (0.99-1.01)	.596	–	–
ALT, U/L	1.00 (0.99-1.01)	.753	–	–
Myocardial Injury Markers				
CK, U/L	1.00 (1.00-1.00)	**.005**	1.00 (1.00-1.00)	.374
CK-MB, U/L	1.00 (0.99-1.00)	.597	–	–
LDH, U/L	1.01 (1.00-1.01)	**<.001**	1.00 (1.00-1.01)	.203
Coagulation Function				
PT, second	1.21 (1.03-1.52)	**.021**	1.19 (0.95-1.42)	.102
APTT, second	1.04 (1.00-1.09)	.069	–	–
D-Dimer, μg/mL	1.17 (1.05-1.34)	**.005**	1.00 (0.58-1.33)	.982
AT3, g/L	0.96 (0.93-0.98)	**<.001**	0.96 (0.94-0.99)	**.004**
Electrolytes				
Potassium, mmol/L	0.57 (0.30-1.10)	.093	–	–
Sodium, mmol/L	1.01 (0.91-1.05)	.649	–	–
Calcium, mmol/L	1.01 (0.96-1.03)	.632	–	–

ALT, alanine aminotransferase; APTT, activated partial thromboplastin time; AST, aspartate aminotransferase; AT3, antithrombin III; BUN, blood urea nitrogen; CK, creatine kinase; CK-MB, creatine kinase-MB; IL-6, interleukin-6; LDH, lactate dehydrogenase; NLR, neutrophil-to-lymphocyte ratio; PICU, pediatric intensive care unit; PT, prothrombin time. Odds ratios and 95% confidence intervals were estimated using univariate Firth logistic regression to account for potential quasi-complete separation. All laboratory parameters were measured upon admission to the PICU.

Bold values indicate statistically significant differences (p < 0.05).

### Multivariate analysis and final prediction model

Multivariate Firth logistic regression identified four independent predictors: fungal co-infection (adjusted OR: 9.01, 95% CI: 2.26–36.49, *P* = 0.003), admission glucose (adjusted OR: 1.23 per mmol/L increase, 95% CI: 1.04–1.48, *P* = 0.022), antithrombin III (adjusted OR: 0.96 per g/L increase, 95% CI: 0.94–0.99, *P* = 0.004), and IL-6 (adjusted OR: 1.00 per pg/mL increase, 95% CI: 1.00–1.01, *P* = 0.008) (**Table 2**). Variables significant in univariate analysis that were not retained included procalcitonin, hemoglobin, ferritin, lactate dehydrogenase, prothrombin time, D-dimer and congenital heart disease (**Supplementary Digital Content 4**).

### Distribution of final predictors

The distribution of the four final predictors revealed distinct clinical patterns between the two groups. Fungal co-infection was nearly eight-fold more frequent in the shock group (25.0% vs. 3.1%). Admission glucose levels in the shock group were notably right-skewed, with several patients exhibiting severe stress-induced hyperglycemia (>11 mmol/L), whereas glucose values in the non-shock group clustered within a narrower, lower range. Antithrombin III levels showed a marked left-shift in the shock group, reflecting early consumptive coagulopathy at the time of PICU admission. IL-6 levels were substantially elevated in the shock group, with a wide interquartile range indicating heterogeneity in the inflammatory response. These patterns underscore the pathophysiological differences captured by the model at the earliest stage of admission.

### Model performance and validation

**Figure 1:**
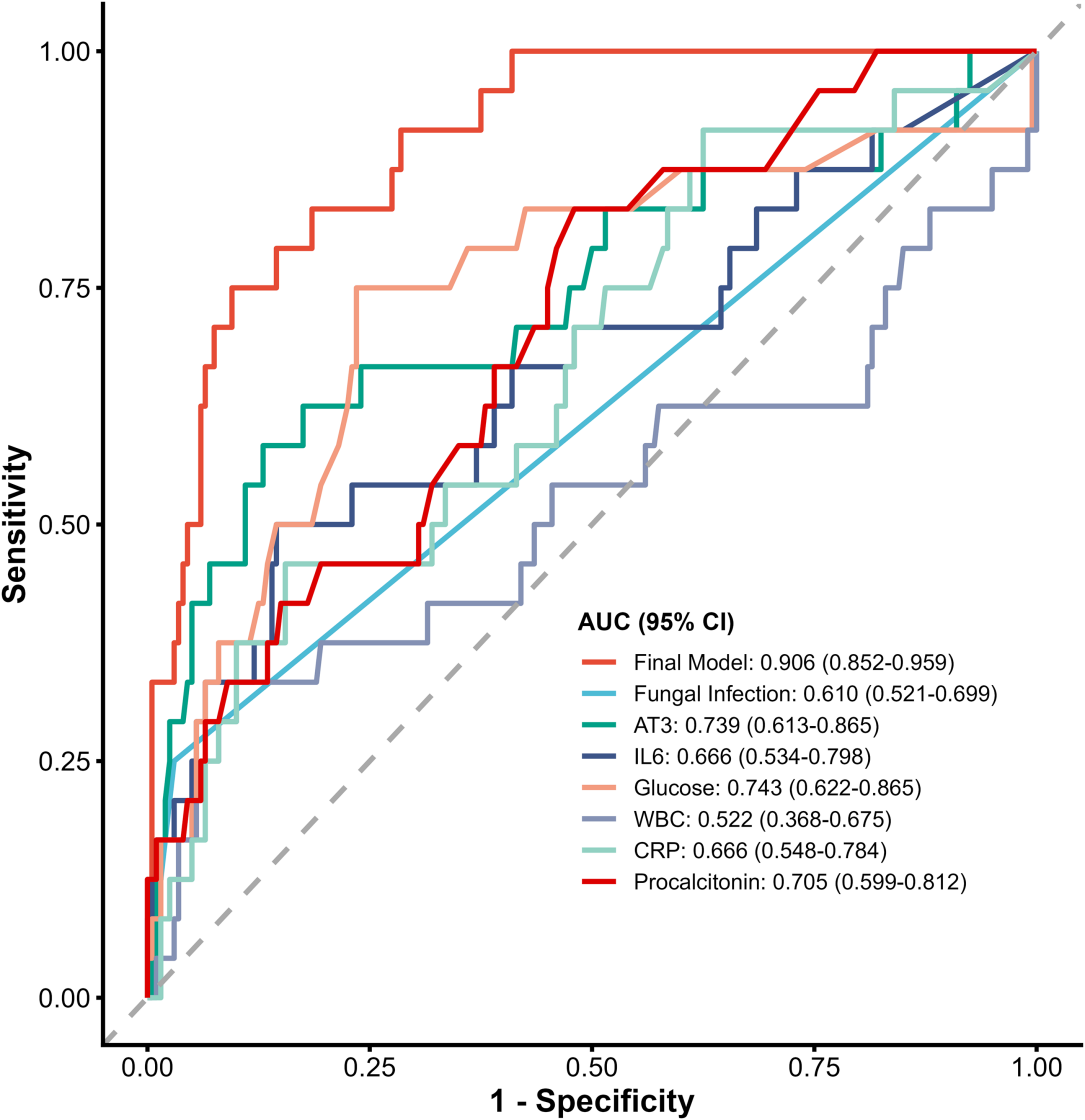
Receiver operating characteristic (ROC) curves comparing the final predictive model and individual candidate biomarkers. The final multivariable predictive model (red line) demonstrated the highest discriminative ability for predicting sepsis shock compared to individual clinical variables and traditional infection markers. The exact area under the curve (AUC) and 95% confidence intervals (CI) for each respective predictor are detailed in the lower right legend. The dashed diagonal line represents the reference line of no discrimination (AUC = 0.50). ROC, receiver operating characteristic; AUC, area under the curve; CI, confidence interval; AT3, antithrombin III; IL-6, interleukin-6; WBC, white blood cell; CRP, C-reactive protein.

The final model demonstrated excellent discrimination, with a mean AUC of 0.892 (95% CI: 0.870–0.914) in 5-fold cross-validation repeated 5 times, and a bias-corrected AUC of 0.906 (95% CI: 0.838–0.949) in bootstrap validation (1,000 resamples). Calibration assessment showed good agreement between predicted and observed outcomes (Hosmer-Lemeshow χ²=5.991, df=8, *P* = .648; Brier score 0.065; calibration intercept 0.002; calibration slope 0.957) (**Figure 1**).

### Sensitivity analysis

(1) Exclusion of Fungal Co-infection: To evaluate the influence of fungal co-infection on model performance, we conducted a sensitivity analysis excluding this predictor. The reduced model, comprising only admission glucose, antithrombin III, and interleukin-6, demonstrated an AUC of 0.837 (95% CI: 0.750–0.924) in bootstrap validation, with a sensitivity of 66.7% and specificity of 87.5% at the optimal threshold of 0.133. Although the performance metrics decreased slightly compared to the original full model (AUC 0.906), the reduced model still exhibited good discrimination and calibration (**Supplementary Digital Content 5**). This finding suggests that while fungal co-infection contributes independently to risk prediction, the remaining three predictors collectively continue to demonstrate clinically useful predictive information.

(2) LASSO Regression Analysis: To evaluate the robustness of our variable selection and mitigate concerns regarding potential overfitting due to the limited number of outcome events, we conducted a LASSO logistic regression analysis (**Supplementary Digital Content 6**). By tracking the coefficient shrinkage path, we identified the four strongest predictors that survived at the maximal penalty. The LASSO-selected model demonstrated good discriminative ability, with an AUC of 0.865 (95% CI: 0.777–0.954) at the optimal threshold of 0.055 (**Supplementary Digital Content 7**). At this threshold, the model exhibited high sensitivity (95.8%) and negative predictive value (99.3%), indicating strong performance in ruling out patients unlikely to develop septic shock. The positive predictive value was modest (26.1%), consistent with the low event rate and the model’s intended role as a screening tool for early risk stratification.

### Clinical performance at optimal threshold

**Figure 2:**
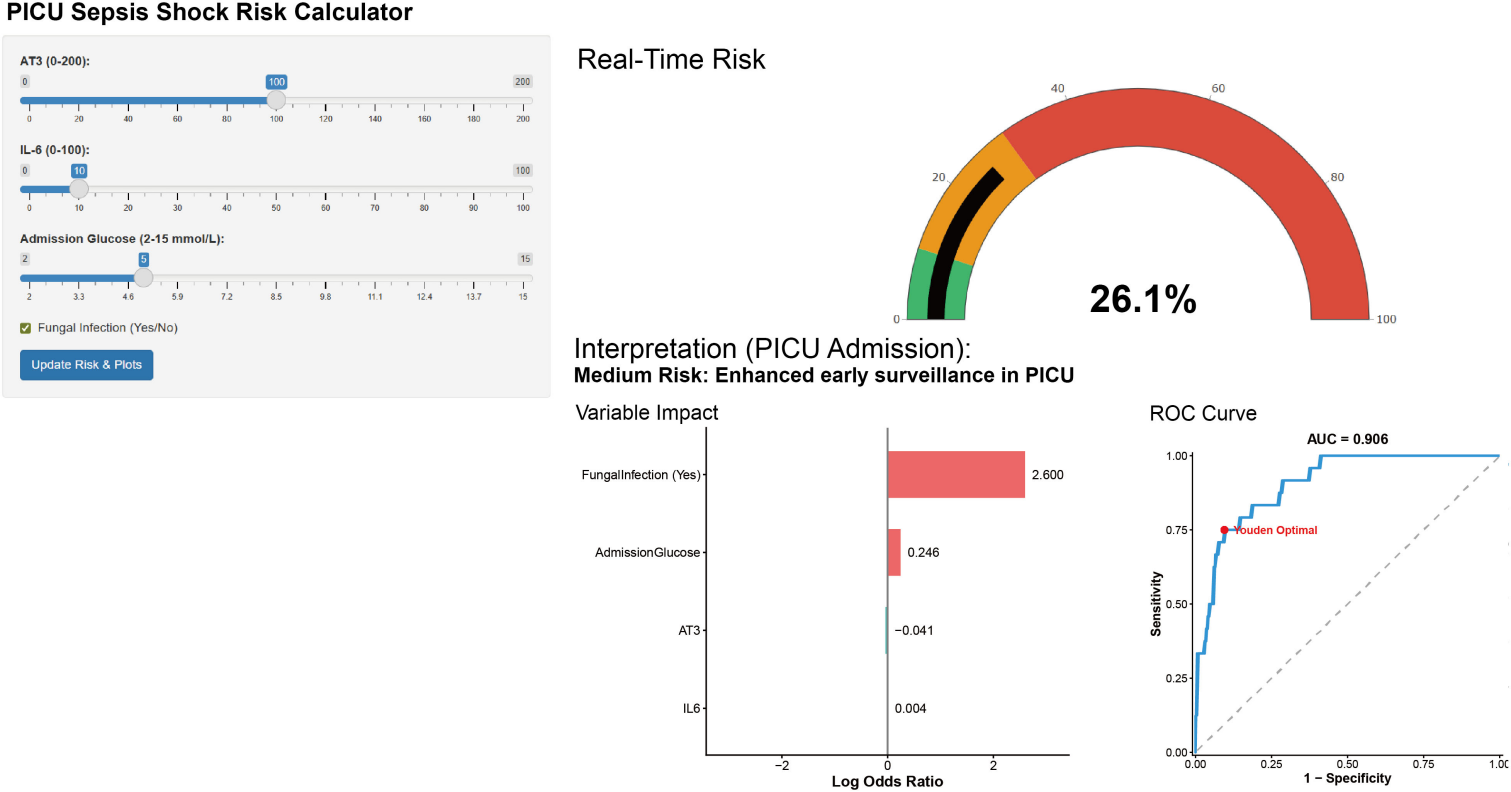
Prototype web-based risk calculator developed using Shiny for the real-time prediction of sepsis shock. The interactive application provides real-time risk stratification for patients admitted to the PICU. The left panel displays input controls for patient-specific clinical parameters, including antithrombin III (AT3), interleukin-6 (IL-6), admission glucose, and fungal infection status. The right panel presents the output dashboard, which includes a gauge chart displaying the real-time probability of sepsis shock, an automated clinical interpretation guide, a horizontal bar chart detailing the relative weight (Log Odds Ratio) of each individual parameter's contribution to the current patient's risk, and the multivariable model's overall ROC curve indicating the Youden optimal threshold. PICU, pediatric intensive care unit; AT3, antithrombin III; IL-6, interleukin-6; OR, odds ratio; ROC, receiver operating characteristic; AUC, area under the curve.

At the probability threshold of 0.154 determined by Youden’s index, the model achieved sensitivity 75.0%, specificity 90.5%, positive predictive value 48.7%, negative predictive value 96.8%, F1 score 59.0%, and overall accuracy 88.8%. This optimal threshold of 0.154 (15.4%) corresponds to the probability above which the model identifies patients as high-risk. At this threshold, the model correctly identifies 75% of patients who will develop septic shock (sensitivity), while correctly classifying 90.5% of those who will not develop shock (specificity). Individually, glucose (cutoff ≥7.45 mmol/L) had an AUC of 0.743, antithrombin III (<83.85 g/L) an AUC of 0.739, IL-6 (≥55.76 pg/mL) an AUC of 0.666, and fungal co-infection an AUC of 0.610. Notably, AT3 demonstrated a high negative predictive value (NPV 94.6%), indicating that preserved AT3 levels at admission effectively rule out the subsequent development of septic shock. The integrated model outperformed all individual predictors (AUC 0.892) (**Table 3**). A web-based risk calculator was developed using the Shiny package in R and published at http://47.109.89.21:3838/clinical_model/ facilitate real-time bedside risk assessment (**Figure 2**).

**Table 3:** Comparative performance of the final predictive model versus individual candidate variables and standard infection biomarkers.

Model	Threshold	AUC (95% CI)	Sensitivity	Specificity	PPV	NPV	F1	Accuracy	Youden index
Final model	0.154	0.906 (0.852-0.959)	0.750	0.905	0.487	0.968	0.590	0.888	0.655
AT3	83.850	0.739 (0.613-0.865)	0.583	0.870	0.350	0.946	0.438	0.839	0.453
Glucose	7.450	0.743 (0.622-0.865)	0.750	0.765	0.277	0.962	0.405	0.763	0.515
IL-6	55.760	0.666 (0.534-0.798)	0.500	0.855	0.293	0.934	0.369	0.817	0.355
Fungal Infection	0.293	0.610 (0.521-0.699)	0.250	0.970	0.500	0.915	0.333	0.893	0.220
WBC	17.480	0.522 (0.368-0.675)	0.333	0.900	0.286	0.918	0.308	0.839	0.233
CRP	12.480	0.666 (0.548-0.784)	0.458	0.845	0.262	0.929	0.333	0.804	0.303
Procalcitonin	0.185	0.705 (0.599-0.812)	0.833	0.520	0.172	0.963	0.286	0.554	0.353

AUC, area under the curve; PPV, Positive Predictive Value; NPV, Negative Predictive Value, F1, F1 Score, AT3, antithrombin III; IL-6, interleukin-6; WBC, white blood cell; CRP, C-reactive protein.

## Discussion

This study developed and validated a novel predictive model for the early identification of septic shock risk among pediatric patients with RSV infection admitted to the PICU. The final model incorporates four readily measurable admission parameters—fungal coinfection, blood glucose level, antithrombin III, and interleukin-6—and demonstrated good discrimination, calibration, and potential clinical utility.

### Interpretation of findings

In this cohort, the occurrence of septic shock was associated with fungal coinfection at admission, hyperglycemia, reduced antithrombin III, and elevated IL-6 levels. These findings are consistent with the established pathophysiology of sepsis: fungal coinfection suggests a more complex microbial milieu, glucose dysregulation reflects the severity of stress response, and disturbances in coagulation and cytokine balance indicate broader systemic instability (Srinivasan, 2012; Kozel and Wickes, 2014; El-Sherbini et al., 2018; Niederwanger et al., 2018; Lestari et al., 2019; Varga et al., 2025). However, these associations do not imply causality or modifiability. Importantly, fungal co-infection should be interpreted primarily as a marker of underlying disease severity or host susceptibility rather than a direct trigger of septic shock (Gatica et al., 2023). Its association with shock may reflect a more complex microbial milieu or greater physiological vulnerability, rather than a modifiable causal pathway. Moreover, the time required for definitive microbiological confirmation may limit its utility as a truly immediate predictor at admission (García-Algar et al., 2025). Each parameter may represent a different facet of sepsis risk in different patients, and the precise interplay among them warrants further elucidation.

The sensitivity analysis excluding fungal co-infection provides further insights into the robustness of our model. Even without this variable, the model retained an AUC of 0.837, indicating that the combination of glucose, antithrombin III, and interleukin-6 alone offers substantial predictive capacity for septic shock. This finding alleviates, at least in part, concerns regarding the practical utility of fungal co-infection as an immediate admission predictor, as the core model remains clinically useful even when fungal status is unavailable or pending confirmation. Nevertheless, the inclusion of fungal co-infection notably improved model performance (AUC increased from 0.837 to 0.906), underscoring its incremental value as a marker of disease severity or host susceptibility. These results reinforce the stability of the variable selection and support the overall validity of our prediction model.

To further evaluate the robustness of our variable selection and address concerns regarding potential overfitting due to the limited number of outcome events, we conducted a LASSO logistic regression analysis as an additional sensitivity measure. By applying L1 regularization, LASSO performs automated variable selection while mitigating overfitting, offering a valuable complement to conventional P-value-based methods. Notably, the predictors selected by LASSO showed substantial overlap with those in our primary Firth regression model. The LASSO-selected model achieved an AUC of 0.865 (95% CI: 0.777–0.954), with high sensitivity (95.8%) and negative predictive value (99.3%) at the optimal threshold of 0.055, while the modest positive predictive value (26.1%) aligns with the low event rate and supports the model’s role as a screening tool. The convergence between these two distinct approaches provides compelling evidence for the stability of our core predictor set, suggesting that the identified associations are unlikely to be artifacts of overfitting. Furthermore, the consistently high NPV across both models supports the potential clinical utility of these predictors in identifying low-risk patients who may be managed with routine monitoring, thereby optimizing PICU resource allocation.

### Clinical performance and threshold interpretation

At the optimal threshold of 0.154 determined by Youden’s index, our model demonstrated a sensitivity of 75.0% and specificity of 90.5%, with a positive predictive value of 48.7%. This indicates that approximately half of the patients identified as high risk may not ultimately develop septic shock. Therefore, the model should be viewed as a screening tool for early risk stratification rather than a diagnostic instrument. For clinicians using the Shiny application, a predicted probability below 15.4% is categorized as ‘Low Risk,’ indicating that the patient is unlikely to progress to septic shock and may be managed with routine monitoring. A probability at or above 15.4% is categorized as ‘High Risk,’ suggesting that the patient warrants closer observation, more frequent vital sign assessments, and consideration of further diagnostic evaluation. Importantly, this threshold is not a diagnostic cutoff but rather a guide for clinical decision-making, and should always be integrated with the clinician’s overall judgment and dynamic patient assessment. In comparison, individual predictors showed inferior performance: glucose (AUC 0.743), antithrombin III (AUC 0.739), IL-6 (AUC 0.666), and fungal co-infection (AUC 0.610). The integrated model (AUC 0.892) substantially outperformed each individual parameter, underscoring the value of combining multiple biomarkers for risk prediction.

### Comparison with previous studies

RSV is widely recognized as a major pathogen of severe lower respiratory tract infection and a common cause of PICU admission in children (Wang et al., 2024). Nevertheless, predictive models specifically targeting the risk of septic shock in pediatric RSV infection remain scarce. Prior research has largely focused on predictors of ICU admission or mortality (Niederwanger et al., 2018; Liu et al., 2025). The main contribution of the present work is the early development of a model tailored to a specific sepsis phenotype—septic shock.

Fungal–bacterial coinfections are not uncommon in patients with severe infections, with substantial prevalence in high-risk populations and increased therapeutic complexity. The association observed here between fungal infection and septic shock may reflect two mechanisms: direct amplification of immunoinflammatory responses or its role as a marker of underlying disease severity or host susceptibility. Importantly, fungal diagnosis is often delayed, with microbiological confirmation typically requiring several days, which limits its utility as an early prognostic indicator (Hage et al., 2019; Zhao et al., 2021; Salazar et al., 2022; Silva et al., 2025).

Stress-induced hyperglycemia is frequently observed in critically ill patients and has been linked to adverse outcomes in various clinical settings. However, blood glucose is a relatively coarse biomarker influenced by multiple factors, including pre-admission feeding status, stress intensity, and iatrogenic effects (Srinivasan, 2012; El-Sherbini et al., 2018). Likewise, reduced AT-III may reflect consumptive coagulopathy, but its independent prognostic value in pediatrics remains uncertain (Niederwanger et al., 2018; Xiang et al., 2021). These parameters should therefore be viewed as indicators of global severity and physiological instability, rather than as direct therapeutic targets.

## Limitations

Despite the promising statistical performance of our model, several important limitations must be acknowledged, with the small sample size representing the most significant barrier to clinical implementation. First and foremost, our analysis included only 24 outcome events (septic shock cases), which substantially increases the risk of overfitting and model instability. This is reflected in the wide confidence intervals observed for key predictors, particularly fungal co-infection (adjusted OR: 9.01, 95% CI: 2.26–36.49). While we employed Firth logistic regression to mitigate small-sample bias, this statistical approach cannot eliminate the intrinsic uncertainty associated with low event rates. Consequently, external validation in large, multicenter cohorts is an absolute prerequisite before any clinical application can be considered (Suhas et al., 2023; D'Angelo and Ran, 2025). Second, all prediction models face the risk of “performance drift,” whereby accuracy declines in different populations (Collins et al., 2024). It remains unproven whether risk stratification based on these four parameters would alter clinical decision-making or improve outcomes and the model’s predictive value must not be misinterpreted as evidence supporting specific interventions such as fungal testing strategies, glycemic targets, or AT-III supplementation. Before implementation, prospective or controlled studies evaluating its impact on clinical behavior are recommended. Third, this was a single-center retrospective observational study, limiting causal inference. The sample size was modest and the incidence of septic shock relatively low, restricting the number of parameters that could be included and potentially leading to omission of important predictors or misestimation of existing effects. The findings may not be directly generalizable to other populations or settings with different case mixes, clinical practices, or fungal epidemiology. Finally, several clinically relevant indicators of organ dysfunction (e.g., SOFA score) and sepsis severity were not included in the model. Lactate, an important marker of tissue hypoperfusion, was not routinely measured at admission in this cohort (>40% missingness), precluding reliable inclusion. Markers of respiratory failure (such as the PaO_2_/FiO_2_ ratio or ventilation mode) were not consistently documented at PICU admission and their inclusion might have introduced selection bias. In addition, vasoactive support requirement formed part of the outcome definition and therefore could not be used as a predictor. The absence of these variables might limit the model’s ability to capture disease severity, and future studies should evaluate whether systematic incorporation of such parameters improves model performance.

## Future directions

This study provides preliminary evidence linking several admission biomarkers with the risk of RSV-related septic shock. These findings are hypothesis-generating rather than practice-changing. External validation in independent cohorts—ideally across multiple geographic and clinical settings—is needed before clinical adoption. Given the inherent limitations of a single-center retrospective design, well-designed multicenter prospective cohort studies are urgently needed to validate our findings across diverse populations and clinical settings. Future studies should also aim to incorporate additional clinically relevant parameters that were not available in our dataset, such as lactate, respiratory failure markers (e.g., PaO_2_/FiO_2_ ratio), and dynamic measures of organ dysfunction, to determine whether they improve model performance or add value beyond routine clinical judgment. Prospective research should evaluate the real-world impact of the model on clinical decision-making and patient outcomes, rather than solely its statistical performance. Intervention trials in high-risk patients should be grounded in existing clinical evidence rather than in the model itself. Ultimately, improving outcomes in RSV-associated septic shock is likely to depend on deeper understanding of sepsis biology and more targeted supportive strategies, rather than reliance on prediction models alone.

## Conclusion

We developed and internally validated a predictive model for early identification of septic shock risk in pediatric patients with RSV bronchiolitis admitted to the PICU. The model, incorporating fungal co-infection, admission glucose, antithrombin III, and interleukin-6, showed good discrimination and calibration in this single-centre cohort and may have potential utility for early risk stratification. However, the small number of outcome events and the resulting risk of overfitting require external validation in larger, multicentre cohorts before clinical implementation. Prospective studies are needed to determine whether use of the model improves patient outcomes.

## Data Availability

The raw data supporting the conclusions of this article will be made available by the authors, without undue reservation.
