# Supplementary material for: Development and internal validation of a clinical prediction model for septic shock in pediatric respiratory syncytial virus bronchiolitis based on routine blood biomarkers and concomitant fungal infection
Source: Front Cell Infect Microbiol. 2026 May 15;16:1743976. doi: 10.3389/fcimb.2026.1743976 (PMC13219336; doi:10.3389/fcimb.2026.1743976)
Supplement: Supplementary file 1 [file DataSheet1.docx]

**Supplemental Digital Contents**

**Supplemental Digital Content 1. Method** Diagnostic Criteria of RSV virus.

**Supplemental Digital Content 2.Figure** Flow chart of enrollment.

**Supplemental Digital Content 3. Table** Description of the treatment of missing data.

**Supplemental Digital Content 4. Table** Unadjusted and Firth-Adjusted Odds Ratios for Septic Shock Based on Admission Parameters.

**Supplemental Digital Content 5.** **Table** Sensitivity Analysis: Model Performance After Excluding Fungal Co-infection.

**Supplemental Digital Content 6.Figure** LASSO regression coefficient paths and cross-validation for variable selection as a sensitivity analysis.

**Supplemental Digital Content 7. Table** Performance of the predictive model derived from LASSO regression.

**Supplemental Digital Content 1. Method** Diagnostic Criteria of RSV virus.

1. Antigen detection: colloidal gold immunochromatographic method: after collection, the nasopharyngeal swab sample is immersed into the tube for extraction for stirring and external squeezing to fully saturate the nasopharyngeal swab, after mixing, the sample is dripped into the test plate, and the result of the test plate is determined after 15 minutes.

2. Nucleic acid detection: according to the genome sequence of the conventional nucleic acid detection method (Reverse transcription polymerase chain reaction, RT-PCR): after the collection of samples placed in the virus transport medium (VTM), 2-8 ℃ storage, transferred to the laboratory within 24h. Nucleic acids were extracted using the Shuoshi SSNP-9600A Automatic Nucleic Acid Extraction Instrument (Jiangsu Shuoshi Biotechnology) for about 20 min, and the extracted nucleic acids were used as templates for downstream PCR analysis. Using Respiratory Syncytial Virus (RSV) Nucleic Acid Detection Kit (Daan Genetics) with real-time fluorescence PCR technology, using the highly conserved region of the coding region of the RSV viral genome as the target region, designing specific primers and fluorescent probes, and amplifying RSV viral RNA by one-step reverse transcription-polymerase chain reaction using a fluorescence quantitative PCR instrument, detecting fluorescence signals and plotting real-time amplification curves to achieve the detection of unknown samples. Detection of unknown samples, amplification time of about 90 minutes.

3. Metagenomic next-generation sequencing (mNGS): Pathogen nucleic acids (such as DNA and RNA) are extracted from the samples, and the extracted nucleic acids are subjected to steps such as reverse transcription, enzyme cleavage, and modification, to construct libraries suitable for sequencing, and then the libraries are sequenced using a high-throughput sequencing platform, which generates a large amount of short sequence data. The library is then sequenced using a high-throughput sequencing platform, which generates a large amount of short sequence data, and the sequencing results are analysed by bioinformatics for quality control, sequence splicing, species identification and functional analysis.

**Supplemental Digital Content 2.Figure** Flow chart of enrollment.

**
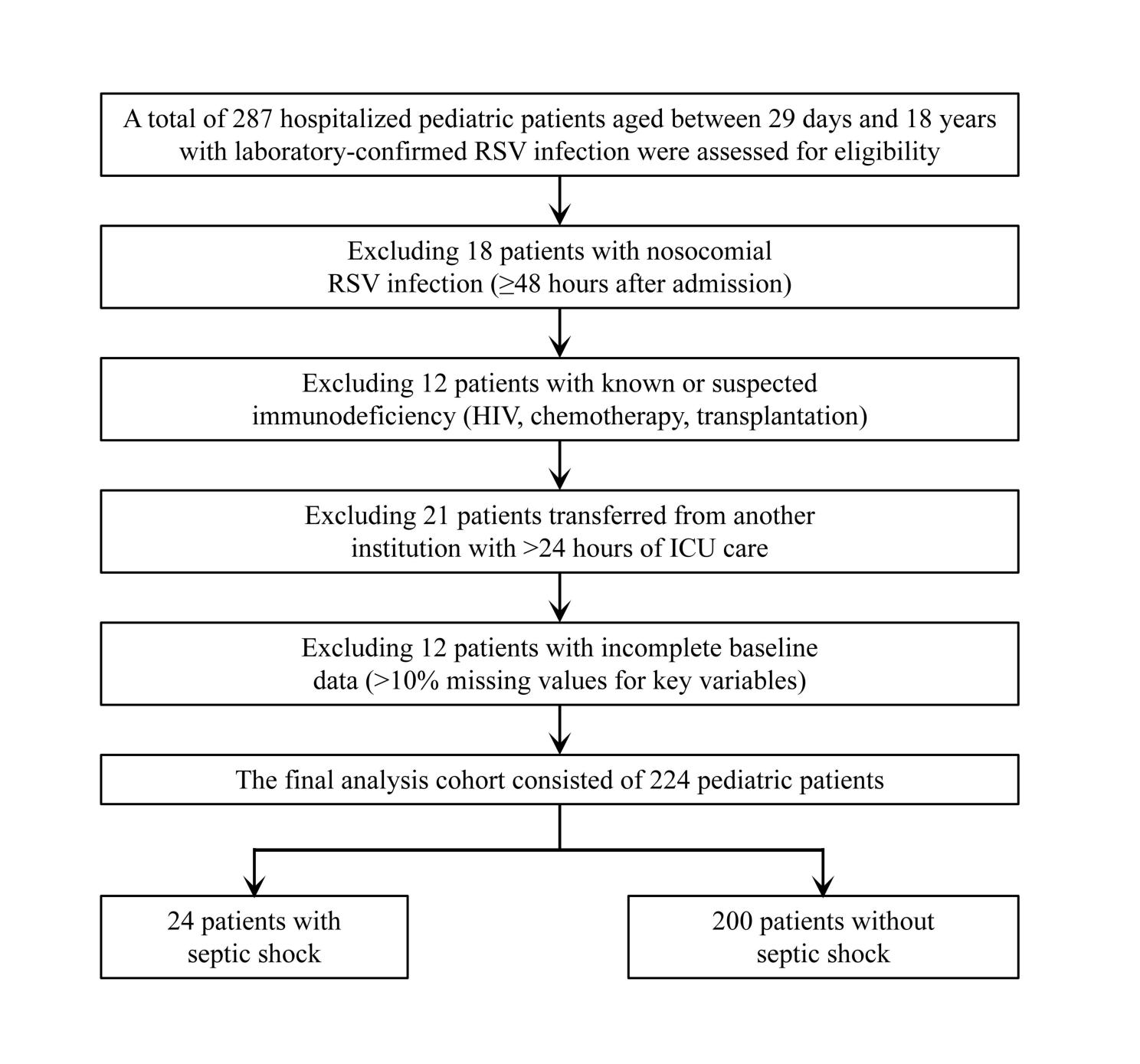
**

**Supplemental Digital Content 3. Table** Description of the treatment of missing data.

|  | **Variable** | **Missing Percent** |
| --- | --- | --- |
| **1** | Glucose | 5.36 |
| **2** | Antibiotics Use | 1.79 |
| **3** | Fungal Infection | 1.79 |
| **4** | Allergy History | 1.79 |
| **5** | Procalcitonin | 1.34 |
| **6** | Ferritin | 1.34 |
| **7** | Drug Sensitivity | 1.34 |
| **8** | IL-6 | 0.89 |
| **9** | Asthma History | 0.45 |
| **10** | Feeding Method | 0.45 |

**Notes:** A total of 224 pediatric patients were included in the analysis. Missing data assessment revealed that the main variables with missing values were glucose (5.36%), fungal infection (1.79%), and procalcitonin (1.34%). Little’s MCAR test (χ² = 5190, df = 5928, P = 1.000) indicated that the missingness mechanism was completely at random (Missing Completely at Random, MCAR). To address missing data, multiple imputation by chained equations (MICE) was performed. The imputation model was automatically selected based on variable type: predictive mean matching (pmm) for continuous variables, logistic regression (logreg) for binary variables, and multinomial logistic regression (polyreg) for categorical variables with more than two levels. A total of five imputed datasets (m = 5) were generated with 50 iterations (maxit = 50), using a fixed random seed (123) to ensure reproducibility. The first completed dataset was extracted for subsequent analyses.

**Supplemental Digital Content 4. Table** Unadjusted and Firth-Adjusted Odds Ratios for Septic Shock Based on Admission Parameters.

| **Variable** | **Unadjusted OR (95%CI)** | ***P* value** | **Adjusted OR (95%CI)** | ***P* value** |
| --- | --- | --- | --- | --- |
| **Fungal Infection** | 10.51 (3.15–35.37) | **<.001** | 9.01(2.26-36.49) | **.003** |
| **Glucose** | 1.33 (1.16-1.54) | **<.001** | 1.23 (1.04-1.48) | **.024** |
| **AT3** | 0.96 (0.93-0.98) | **<.001** | 0.96 (0.94-0.99) | **.004** |
| **IL-6** | 1.00 (1.00-1.01) | **<.001** | 1.00 (1.00-1.01) | **.008** |
| Procalcitonin | 1.24 (1.07-1.58) | **<.001** | 0.98 (0.88-1.33) | .723 |
| Hemoglobin | 0.94 (0.91-0.97) | **<.001** | 0.97 (0.93-1.00) | .082 |
| Ferritin | 1.00 (1.00-1.01) | **.001** | 1.00 (1.00-1.00) | .833 |
| LDH | 1.01 (1.00-1.01) | **.001** | 1.00 (1.00-1.01) | .202 |
| PT | 1.21 (1.03-1.52) | **.021** | 1.19 (0.95-1.42) | .104 |
| D-Dimer | 1.17 (1.05-1.34) | **.005** | 1.00 (0.58-1.33) | .981 |
| CK | 1.00 (1.00-1.00) | **.005** | 1.00 (1.00-1.00) | .374 |
| Congenital Heart Disease | 3.62 (1.01-11.25) | **.005** | 1.82 (0.21-12.40) | .563 |

**Abbreviations:** PICU, Pediatric Intensive Care Unit; IL-6, Interleukin 6; CK, Creatine Kinase; LDH, Lactate Dehydrogenase; PT, Prothrombin Time; AT3, Antithrombin III.

**Supplemental Digital Content 5. Table** Sensitivity Analysis: Model Performance After Excluding Fungal Co-infection.

| **Model** | **Threshold** | **AUC (95%CI)** | **Sensitivity** | **Specificity** | **PPV** | **NPV** | **F1** | **Accuracy** | **Youden Index** |
| --- | --- | --- | --- | --- | --- | --- | --- | --- | --- |
| Original full model (with fungal co-infection) | 0.154 | 0.906 (0.852-0.959) | 0.750 | 0.905 | 0.486 | 0.968 | 0.590 | 0.888 | 0.655 |
| Model excluding fungal co-infection | 0.133 | 0.837 (0.750-0.924) | 0.667 | 0.875 | 0.390 | 0.956 | 0.492 | 0.853 | 0.542 |

**Abbreviations:** AUC, area under the curve; PPV, Positive Predictive Value; NPV, Negative Predictive Value, F1, F1 Score.

**Supplemental Digital Content 6. Figure** LASSO regression coefficient paths and cross-validation for variable selection as a sensitivity analysis.


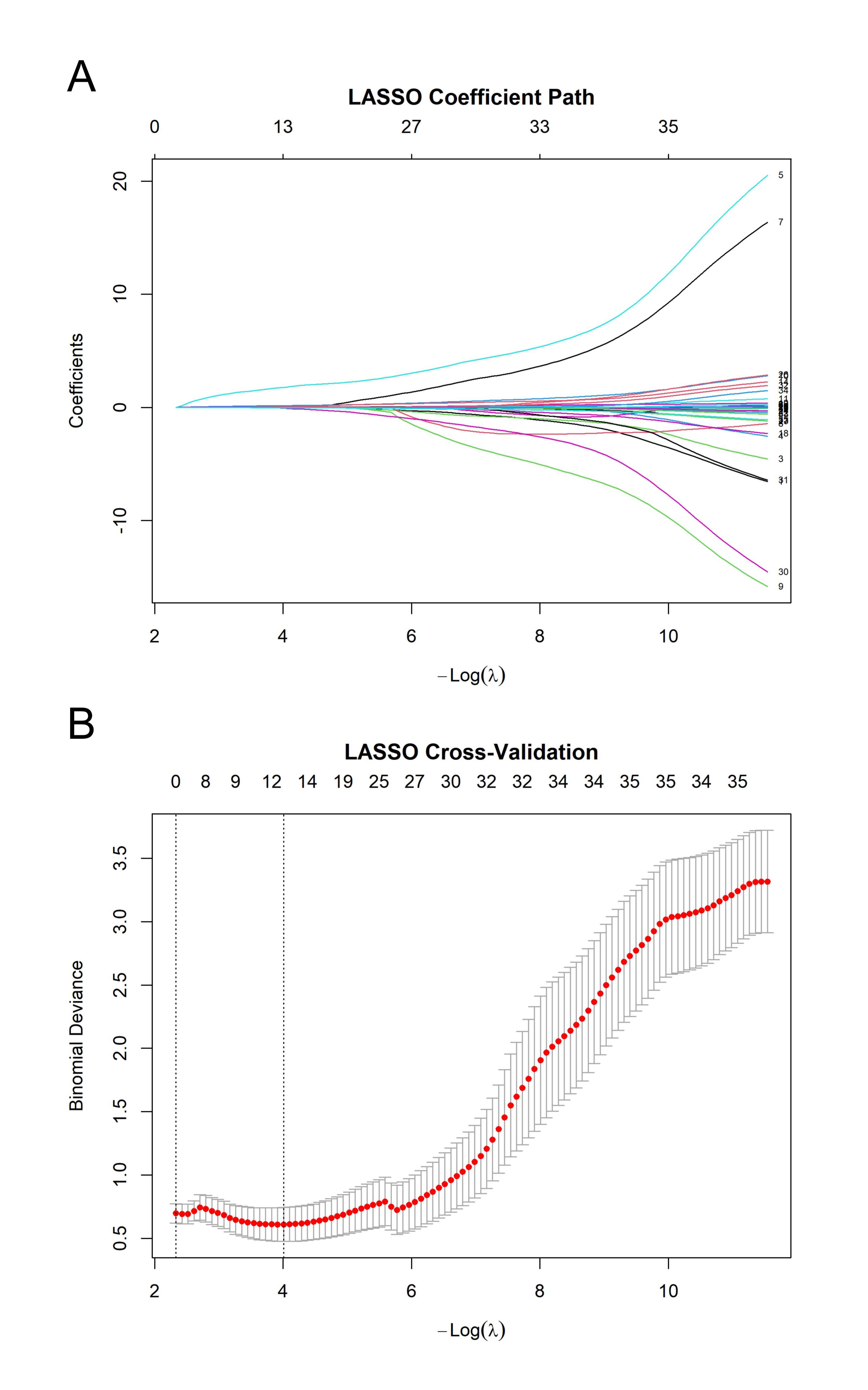


**Supplemental Digital Content 7. Table** Performance of the predictive model derived from LASSO regression.

| **Model** | **Threshold** | **AUC (95%CI)** | **Sensitivity** | **Specificity** | **PPV** | **NPV** | **F1** | **Accuracy** | **Youden Index** |
| --- | --- | --- | --- | --- | --- | --- | --- | --- | --- |
| Lasso | 0.055 | 0.865 (0.777-0.954) | 0.958 | 0.675 | 0.261 | 0.993 | 0.411 | 0.705 | 0.633 |

**Abbreviations:** AUC, area under the curve; PPV, Positive Predictive Value; NPV, Negative Predictive Value, F1, F1 Score.
